# Micro- and Macroelements Content of Plants Used for Landfill Leachate Treatment Based on *Phragmites australis* and *Ceratophyllum demersum*

**DOI:** 10.3390/ijerph19106035

**Published:** 2022-05-16

**Authors:** Aleksandra Wdowczyk, Agata Szymańska-Pulikowska

**Affiliations:** Institute of Environmental Engineering, Wroclaw University of Environmental and Life Sciences, pl. Grunwaldzki 24, 50-363 Wroclaw, Poland; agata.szymanska-pulikowska@upwr.edu.pl

**Keywords:** municipal solid waste (MSW), landfills leachate treatment, phytoremediation, bioconcentration factor (BCF), translocation factor (TF)

## Abstract

One of the key problems associated with the functioning of landfills is the generation of leachate. In order to reduce their negative impact on the environment, various treatment technologies are applied. Among them, solutions based on the use of phytotechnology deserve special attention. The aim of this study was to evaluate the impact of landfill leachate on the content of micro- and macroelements in plant material. The research was carried out in four municipal waste landfills located in Poland. Emergent macrophytes (*P. australis*) and submergent macrophytes (*C. demersum*) were used in this research. The migration and distribution of pollutants reaching the roots and shoots of *P. australis* from water solutions were also studied. The concentrations of heavy metals in the studied plants were low in all analysed cases. Higher metal contents could often be observed in roots rather than in shoots, but these differences were insignificant. The chemical composition of the studied plant samples was primarily related to the source of origin of the treated leachate (landfill), as clearly demonstrated by cluster analysis. In the conducted studies, no important differences were noted in the accumulation of the studied components between submergent plants (*C. demersum*) and emergent macrophytes (*P. australis*).

## 1. Introduction

The landfilling of municipal waste is a common waste management practice [1,2]. One of the key problems associated with the functioning of landfills is the generation of leachate [3]. Leachate may be a potential environmental hazard due to the high content of inorganic compounds and dissolved organic compounds, suspended solids, heavy metals and hazardous substances [4,5,6,7,8]. The most frequent pollutants in leachate are ammonium nitrogen, chlorides, sulphates and heavy metals. In natural ecosystems, heavy metals are characterised by high mobility, relatively high chemical stability and carcinogenicity [9]. Less common are humic acids, dioxins, furans, pesticides, phthalates or pharmaceutical compounds [10,11,12,13]. Long-term contact with the substances contained in leachate may result in their uptake by aquatic organisms and bioaccumulation in subsequent links of the food chain [14,15]. Migration in the environment may also lead to serious threats to human health [16].

In order to reduce the negative impact of landfill leachate on the environment, various treatment technologies are applied. Due to the complexity and variety of leachates, as well as the disadvantages of the technologies used, despite many years of research, there is still no agreement as to the optimal method of their treatment [17].

Among the methods used to treat leachate from landfills, biological, chemical and physical ones can be distinguished. Physical and chemical methods (e.g., coagulation/flocculation, membrane treatment or chemical precipitation) are recommended mainly in the treatment of mature leachate with low biodegradability [18]. However, biological methods are recommended for young leachates due to their high biodegradability. Biological processes are preferred for the treatment of landfill leachate, but it should be taken into account that they have their limitations and do not always provide the expected effectiveness in the treatment of mature leachate [19].

Among them, solutions based on the use of phytotechnology deserve special attention [20,21]. Phytoremediation is used to eliminate the pollutants present in leachate, including organic compounds (petroleum hydrocarbons, pesticides) and trace elements (metals, metalloids), in water and soil [22]. It is a set of techniques or processes in which plants are used to extract, store, degrade or immobilise pollutants from a site (soil, water or sediment) [23].

Depending on the mechanisms used by plants, phytoremediation may include: phytodegradation (the decomposition of pollutants), phytoextraction (the accumulation of pollutants in plant tissues) and phytostabilisation (the transformation of toxins into non-toxic or less toxic forms that leads to the reduced mobility of pollutants through accumulation in roots or immobilisation in the rhizosphere) [22,24,25].

Phytoremediation is considered to be an effective, low-cost and environmentally friendly pollutant removal technology. Some plants have the ability to take up and bind biogenic compounds and metals in less harmful forms in tissues that are metabolically inactive [26]. Differences in the efficiency of the extraction of nutrients and metals from wastewater can be observed among different plant species [27,28]. Constructed wetlands are a well-known technology for wastewater treatment. They are recognized to be effective in the removal of dissolved organics and suspended solids [29].

To date, constructed wetlands have usually been planted with Phragmites spp. (Poaceae), Scirpus spp. (Cyperaceae), Iris spp. (Iridaceae), Typha spp. (Typhaceae), Eleocharis spp. (Cyperaceae), Juncus spp. (Juncaceae) and Salix [30] worldwide. Unfortunately, most plant species act selectively and do not show efficiency in removing pollutants from multi-component mixtures [31]. Therefore, the selection of suitable plant species is a very important issue, as they should survive the potentially toxic effects of the pollutants present in leachate and their variability over time [31]. In addition, these plants should have the ability to produce large amounts of biomass and accumulate high concentrations of pollutants [32].

Recently, the most frequently used aquatic plant in phytoremediation has been *Phragmites australis* [33], which is one of the most widely distributed emergent plant species in the world. It is commonly found in Europe but also in North America and various regions of South America and Australia [34,35]. In addition to being a species with a very wide geographical range, its advantages include its low cost and minimal requirements [36]. *P. australis* is not a hyperaccumulator, but it is characterised by a fast growth rate, high biomass production and a deep root system [37]. All these characteristics make it one of the favourite test species and therefore, since the 1970s, it has been widely used in the phytoremediation of different types of wastewaters, soils and sediments [38]. The efficiency of *P. australis* in the bioaccumulation of heavy metals and other pollutants has been confirmed in numerous studies [37,39,40].

Among aquatic plants, submergent macrophytes are also considered very effective in the phytoremediation of pollutants [41]. They are among the major primary producers in aquatic ecosystems and, due to their large contact area, they show a higher potential for removing pollutants from liquids [42,43]. *Ceratophyllum demersum* is one of the representatives of submergent macrophytes, common throughout the world. It has no roots but can anchor itself by means of rhizoids—modified root-like leaves [44]. The conducted studies show that *C. demersum* is able to produce an internal concentration of metals and other components several times higher than in the surrounding environment. The high biomass growth, high removal efficiency and wide range of tolerance to metals make these plants an excellent choice for the phytoremediation process [45,46].

To date, a large amount of research has been carried out on leachate treatment using phytoremediation. However, to the best of our knowledge, few studies conducted so far have included analyses of the content of micro- and macroelements in the context of the effectiveness of their collection from leachate from landfills. Moreover, after analysing the literature, the authors observed that few studies compared the effectiveness of phytoremediation with the use of emerged and submerged plants on leachate from various landfills, taking into account the effect of the applied dose of the leachate.

Therefore, this paper will present the range of content and movement of macro- and microelements in the plant material of *Phragmites australis* and *Ceratophyllum demersum*. The plants were exposed to different concentrations of leachate from municipal waste landfills at different stages of operation (active and closed). The first part of the study included the evaluation of the leachate treatment efficiency by *P. australis* and *C. demersum* using physicochemical property analyses and toxicity tests [47]. The results presented here complement the picture of the components of the experimental system by presenting data on micro- and macroelement concentrations in plants.

The aim of this study was to evaluate the impact of landfill leachate on the content of micro- and macroelements in plant material. Emergent macrophytes (*P. australis*) and submergent macrophytes (*C. demersum*) were used in this research. The migration and distribution of pollutants reaching the roots and shoots of *P. australis* from water solutions were also studied.

## 2. Materials and Methods

### 2.1. The Study Location and Leachate Collection Points

**Figure 1:**
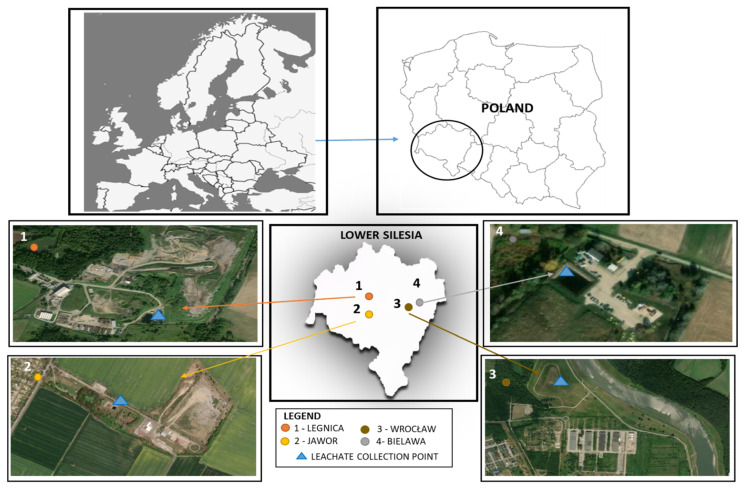
Location of landfills and distribution of leachate collection points.

This study was conducted at four municipal waste landfills, situated in Poland, in the Lower Silesian Voivodeship. Leachate samples were collected in June 2020. Two non-operational landfills (located in Wrocław and Bielawa) and two operational landfills (located in Legnica and Jawor) were selected for the study. Figure 1 shows the location of the leachate collection points.

The oldest landfill site was established in Wrocław (51°10′23.784″ N 16°55′40.74″ E) in 1966 and operated until 2000. It has an area of 11.7 ha and a capacity of about 2 million m^3^. The second disused landfill is located in Bielawa (51°9′21.485″ N 17°14′18.03″ E). The facility was in operation from 2001 to 2011. The surface area of the landfill is 0.86 ha and its capacity is 37.8 thousand m^3^. The largest operational landfill is located in Legnica (51°14′21.317″ N 16°11′0.251″ E), with an area of 14.12 ha and a total capacity of 2.34 million m^3^. The landfill has been in operation since 1977. The second active landfill in Jawor (51°3′56.112″ N 16°12′38.927″ E) has been in operation since 1997. The area occupied by waste is 3.37 ha. The total capacity of the landfill is 231.3 thousand m^3^. The exact characteristics of the landfills where the research was conducted are presented in the publication [10].

### 2.2. Physicochemical Composition of Leachate

Analyses of the physicochemical properties of the leachate were performed at the Environmental Research Laboratory of the Institute of Environmental Engineering at Wrocław University of Environmental and Life Sciences. The studies were performed using commonly used methods, in accordance with ISO (International Organization for Standardization) standards. Laboratory analyses not requiring mineralisation of the samples were performed within 24 h of their collection [48], followed by the analyses requiring mineralisation.

The range of research of the raw and post-treatment by *Phragmites australis* and *Ceratophyllum demersum* included: pH, electrical conductivity (EC), total Kjeldahl nitrogen (TKN), organic nitrogen (ON), ammonium nitrogen (AN), total phosphorus (TP), Chemical Oxygen Demand (COD), biochemical oxygen demand (BOD_5_) and concentrations of: total dissolved solids (TDS), total suspended solids (TSS), total solids (TS), chlorides, sulphates, potassium, sodium, magnesium, calcium, iron, manganese, nickel, zinc, cadmium, copper, lead and chromium.

### 2.3. Conditions of Conducting an Experiment with Phragmites australis and Ceratophyllum demersum

Laboratory tests involved the exposure of *C. demersum* (rigid hornwort), *P. australis* (common reed) and seedlings to increasing concentrations of landfill leachate. The experiment was designed to check the accumulation of micro- and macronutrients and heavy metals in the plant material. The migration and distribution of pollutants from the aqueous solutions to the roots and shoots of *P. australis* were also studied.

The transferred *P. australis* and *C. demersum* plants were flooded with tap water for a period of 14 days to adapt to laboratory conditions. Then, 60 plants of similar sizes were selected from the *P. australis* seedlings that had acclimatised, and they were relocated individually to 1.5 dm^3^ containers. From the *C. demersum* seedlings, 120 plants with an average length of about 20 cm were selected. In each of the 60 containers with a volume of 0.5 dm^3^, two plants were placed.

The containers thus prepared (with the *P. australis* and *C. demersum* seedlings) were completed with the landfills leachate from the four facilities. The series for each landfill consisted of solutions with increasing concentrations: from 0% (tap water), through 6.25%, 12.5%, 25% and 50% to 100% [49]. The leachate exposure lasted for another 14 days [32,50,51]. No additional aeration was applied during the experiment. Each variant was performed in 3 repetitions. The study included an evaluation of the efficiency of the leachate treatment by *P. australis* and *C. demersum* using physicochemical analyses and toxicity tests and is detailed in the publication [52].

### 2.4. Analysis of Selected Components in the Plant Material

After completing the first part of the experiment, the plants were removed from the prepared leachate solutions. *P. australis* was separated into the aboveground part (the stem and leaves) and the underground part (the roots).

The collected plants were dried at room temperature until they reached a constant weight [53]. The dried mass was ground in a laboratory grinder and subjected to chemical analyses. The samples were analysed for: total nitrogen (TN), total phosphorus (TP), sodium, potassium, calcium, magnesium, iron, manganese, zinc, lead, nickel, cadmium, copper and chromium. The results of the analyses are given in mg/g d.m.

The content of the macro- and microelements and heavy metals in the plant material was determined after wet digestion in a mixture of concentrated perchloric acid, sulphuric acid and nitric acid (4:1:10 ratio) [54]. All analyses were performed in three repetitions. The control samples (plants placed in tap water) were subjected to the same procedure.

### 2.5. Bioconcentration Factor (BCF) and Translocation Factor (TF)

The bioconcentration factor (BCF) is used to characterise the behaviour of a chemical substance in the environment [55]. In particular, the BCF is used to verify the ability of a plant to accumulate elements from the substrate or the external solution [56]. A higher BCF value indicates a better phytoaccumulation capacity. The BCF was calculated as follows [46,57]:

(1) $$\mathrm{BCF}=\frac{C_{p}}{C_{w}}$$

where:

C_p_—the pollutant concentration in the plant (mg/kg),
C_w_—the concentration in the external solution (mg/dm^3^).

In calculating the BCF values, the results of the composition analyses of the analysed plants were converted from mg/g to mg/kg.

Apart from checking the ability of the plant to accumulate elements from the substrate or the external solution, it is important to verify the ability to transfer pollutants from the underground parts of the plant to the aboveground parts. It can be characterised by the translocation factor (TF), which is the quotient of the concentration of the pollutants accumulated in the aboveground parts of the plant to the concentration present in the underground parts:

TF = C_a_/C_u_ (2)

where:

C_a_—the pollutant concentration in the aboveground tissues (mg/kg, mg/g),
C_u_—the pollutant concentration in the underground tissues (mg/kg, mg/g).

TF values above 1 indicate the translocation ability [1].

### 2.6. Data Treatment and Statistical Analysis

The obtained results from the plant material, i.e., *Phragmites australis* and *Ceratophyllum demersum*, were subjected to statistical analysis, using Statistica 13.1 software (StatSoft Polska, StatSoft, Inc., Tulsa, OK, USA). Because different concentrations of the leachate were used in the experiment, the chemical composition of the plants was presented in the form of basic descriptive statistics (minimum and maximum).

The assessment of similarity between the plant samples was performed using cluster analysis. It is one of the methods of multivariate analysis, useful in the case of large amounts of data. Hierarchical cluster analysis allows for grouping observations into clusters. Similar observations are placed inside clusters and different clusters contain observations that differ from each other. Clustering is performed on the basis of similarity or distance (dissimilarity). Clusters are aggregated according to decreasing degrees of similarity (or increasing degrees of dissimilarity) until an individual, single cluster with a tree-like structure, called a dendrogram, is formed [58].

The clustering of observations (agglomeration) was performed using Ward’s method (the minimisation of the increase in sum of squares MISSQ). This method is based on minimising the heterogeneity (variance) in clusters and finding the highest possible similarity between observations. It has been shown in many studies to be accurate and useful for creating a primary cluster structure [59,60]. The distances between objects were determined based on the Euclidean distance.

## 3. Results and Discussion

### 3.1. Content of Selected Macro- and Microelements in Landfill Leachate and Plants

#### 3.1.1. Physicochemical Composition of Leachate from Landfills

Table 1 presents the results of the physicochemical analyses of the raw leachate collected from the active and non-operational landfills.

The pH value of the leachate from all landfills ranged from 8.11 (Jawor) to 8.84 (Legnica). An alkaline reaction of the leachate is characteristic of older and mature facilities (i.e., operating for more than 10 years) [61,62], which is the case for the studied leachate.

pH changes could provide a more or less favourable environment for plant growth, and this will vary with the species of the plants [63].

In Europe, the pH of the solid substrate on which *P. australis* grows is generally seven but plants also develop well in a pH range of 5.5 to 7.5 [64]. It has been shown that *P. australis* can tolerate a wide pH range even between 2.5 and 9.8 [65]. *C. demersum* thrives in neutral and alkaline environments, but a pH that is too high can lead to stress in plants. Gao et al. showed that plants experience stress at a pH above nine and with an elevated nitrogen content [66].

The highest EC values were recorded at the active landfills, ranging from 3919 μS/cm (Jawor) to 7794 μS/cm (Legnica). In non-operational landfills, the values were lower and ranged from 1800 μS/cm (Wrocław) to 2318 μS/cm (Bielawa). The EC is related to the amount of ions available to the plants in the root zone. The optimum EC value depends on the species and on the environmental conditions. A high EC value hinders nutrient uptake by increasing the osmotic pressure of the nutrient solution, while an EC concentration that is too low can negatively affect plant health and yield [67,68]. As shown by Huang et al., the pH and EC of the external substrate have a significant effect on nutrient accumulation in plants [69].

Nitrogen is one of the most important nutrients for plants [69] but its too high levels can disrupt the dynamic balance of the antioxidant mechanism, causing the accumulation of reactive oxygen inside plant cells [66,70]. High nitrogen contents were recorded in the leachate from the active landfills (from 269.85 mg/dm^3^ to 310.16 mg/dm^3^). In non-operational landfills, these values were much lower: from 1.09 mg/dm^3^ (Wrocław) to 51.12 mg/dm^3^ (Bielawa).

Phosphorus is essential for plant physiological functions. It plays an important role in key processes such as photosynthesis, respiration, storage and the transfer of energy. It also improves the yield quality and is essential for seed formation [71]. The phosphorus content in the leachate from the active landfills were higher than those from non-operational ones and ranged from 0.22 mg/dm^3^ (Wrocław) to 12.04 mg/dm^3^ (Jawor).

The sodium and potassium content in the samples from the active landfills were also higher than those in the leachate from non-operational landfills. The sodium concentration in the active landfills reached 177.8 mg/dm^3^ (Legnica), whereas in closed ones the values were lower, up to 151.8 mg/dm^3^ (Bielawa). Potassium concentrations were similar; the highest values were observed in the active landfills (up to 507.6 mg/dm^3^ in Legnica), whereas in the closed landfills, the maximum value was recorded in the Bielawa landfill (256.2 mg/dm^3^). These ions play an important role in plant physiology [72].

The Fe and Mn content in the closed landfills were low and, in all cases, amounted to <1 mg/dm^3^. In the active landfills, the values were slightly higher. The Mn content ranged up to 1.61 mg/dm^3^ (Jawor), while the Fe content ranged from 2.16 mg/dm^3^ (Legnica) to 3.81 mg/dm^3^ (Jawor). Fe and Mn in appropriate concentrations have positive effects on plants. Manganese is an important component of various metabolic enzymes [73], and Fe is needed by enzymes to catalyse reactions in the cytochrome [74].

The content of the heavy metals in the leachate from the studied landfills remained at a very low level (i.e., <1 mg/dm^3^), which may indicate that the landfilled waste was mainly municipal waste (not containing these components) or that they may have already leached from the landfilled waste. The analyses performed confirm that heavy metal content is not currently the most serious problem in the management of landfill leachate [5,72,75]. Heavy metals in high concentrations can cause toxic effects in plants, accumulate in aquatic organisms and move up the food chain [76,77]. However, the presence of metals in appropriate concentrations can also have positive effects on plant growth and development [78]. Cu plays an important role in the composition of enzymes for protein synthesis and photosynthesis. Although Cr is not an essential element for plants, there are studies indicating that, at low concentrations, it can have a positive effect on growth stimulation in plants [79].

#### 3.1.2. Chemical Composition of Plants

**Figure 2:**
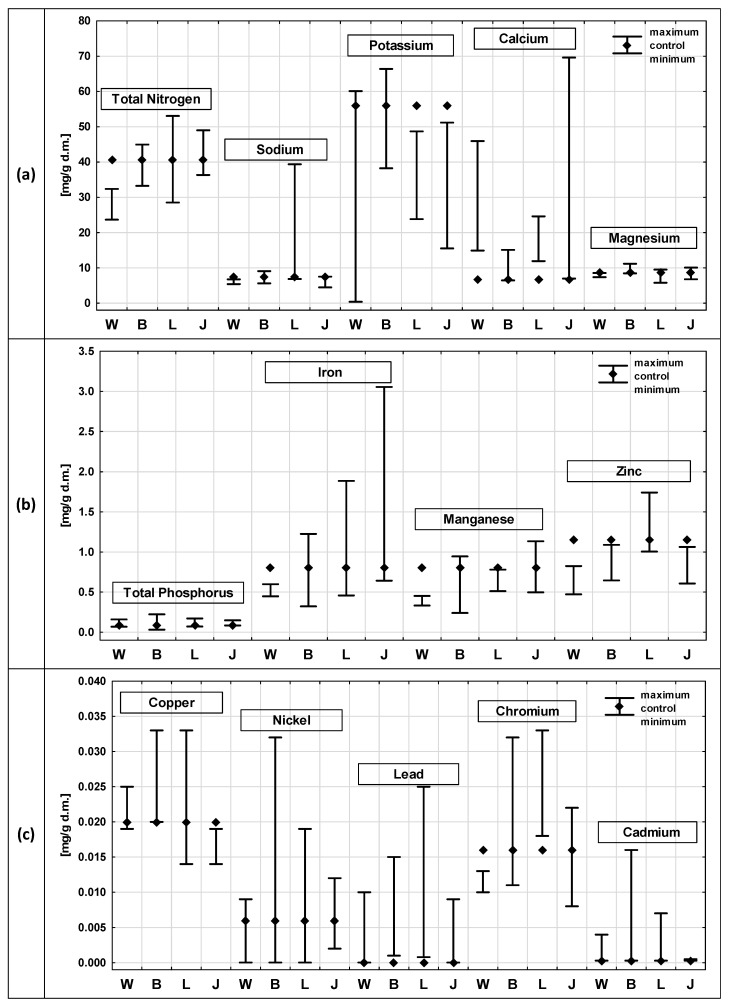
Comparison of chemical composition of plants after exposure to leachate from active (L—Legnica, J—Jawor) and closed (W—Wrocław, B—Bielawa) landfills with the composition of the control sample—results of analyses for *Ceratophyllum demersum*: (**a**) contents of TN, sodium, potassium, calcium and magnesium; (**b**) contents of TP, iron, manganese and zinc; (**c**) contents of copper, nickel, lead, chromium and cadmium.

The results of the chemical composition analysis of *C. demersum* after exposure to the leachate from the four municipal waste landfills and in the control sample are shown in Figure 2. The following parameters were analysed: TN, TP, sodium, potassium, calcium, magnesium, iron, manganese, zinc, lead, nickel, cadmium, copper and chromium.

*C. demersum* is a nitrophile that tolerates high nitrogen concentrations and has a very good nitrogen removal effect; therefore, it is often used for nitrogen removal from wastewater [80]. Only in the case of the plants after exposure to the leachate from the Wrocław landfill were lower TN contents found than those found in the control sample. In the remaining cases, the TN content of the control sample was within the content range corresponding to the plants after contact with the leachate. The TN values ranged from 23.7 mg/g (Wrocław) to 53.1 mg/g (Legnica).

The Na content in the tissues of *C. demersum* remained at a similar level, both in the control sample and after exposure to the leachate. The obtained results did not show any major fluctuations; only in the case of exposure to the leachate from Legnica, the maximum Na content in the plant was significantly higher (amounted to 39.4 mg/g), with the minimum content similar to the other landfills and the control sample. Additionally, the magnesium and TP content in the plant samples after exposure to the leachate from the landfills included in the study did not show marked differences. The contents found were also similar to the results of the control sample.

The potassium content in the *C. demersum* tissues showed rather high variability. The maximum values were higher than those found in the control sample in the case of the Wrocław (60.1 mg/g) and Bielawa (66.4 mg/g) landfills. However, in the case of the plants exposed to the leachate from the landfills in Legnica and Jawor, all the values were lower than those found in the control sample.

The calcium content in the *C. demersum* tissues in most of the analysed cases exceeded those in the control sample. Only the lowest values for the landfills in Bielawa and Jawor were at the level of the control sample. Similar relations are visible in case of the lead and cadmium content. Most of the samples showed an increase in the content of those metals after exposure to the leachate; only minimal values were similar to those of the control sample.

The iron content in the studied plant samples showed quite high variability. The samples after exposure to the leachate from Jawor were characterised with the highest values. Additionally, the majority of the samples from Legnica and Bielawa contained more iron than the control sample. Only the samples after exposure to the leachate from the Wrocław landfill were characterised by a low iron content, below the value for the control sample. Similar relationships were evident for the contents of manganese and chromium (whose highest contents were found in the plants after exposure to the leachate from the landfill in Legnica).

The zinc contents of the tested plants were different. They were higher than the control sample only after the exposure to the Legnica leachate. In the case of copper, however, the contents were lower than those in the control sample only after the exposure of the plants to the leachate from the Jawor landfill. On the other hand, the nickel contents in the control sample were within the range of the values obtained for the samples after contact with the landfill leachate. The highest nickel contents were found in the landfill in Bielawa.

The contents of the majority of the analysed heavy metals (Cu, Ni, Pb, Cr and Cd) did not exceed the level of 1 mg/g. The highest contents of Cu, Ni, Pb, Cr and Cd were recorded after exposure to the leachate from the Legnica and Bielawa landfills and were higher than the values found in the control samples. The data presented in the literature also show that *C. demersum* is able to accumulate significant amounts of various metals, including: Mn, Cu, Cr, Fe, Cd and Pb [57].

The bioavailability of heavy metals is influenced by various factors, especially pH and redox potential [81]. During the conducted studies, it was found that metal concentrations in water and soil tended to be negatively correlated with pH, which affects the amount of metals taken up by plants [82] and may explain the rather low heavy metal content in the plants studied.

In the case of the TP, TN, Na, K, Ca, Mg and Cd contents, in each variant in the external solution, higher contents were observed than in the *C. demersum* plants. The reverse was true for Zn, where higher concentrations were observed in *C. demersum* than in the external solution. Additionally, others, during studies on *C. demersum,* have observed that they are able to produce internal concentrations of metals and other components several times higher than the surrounding environment [45,46]. For Fe, Mn, Cu, Ni, Pb and Cr, the situation was variable once higher values were recorded in the external solution and once they were in the plants.

**Figure 3:**
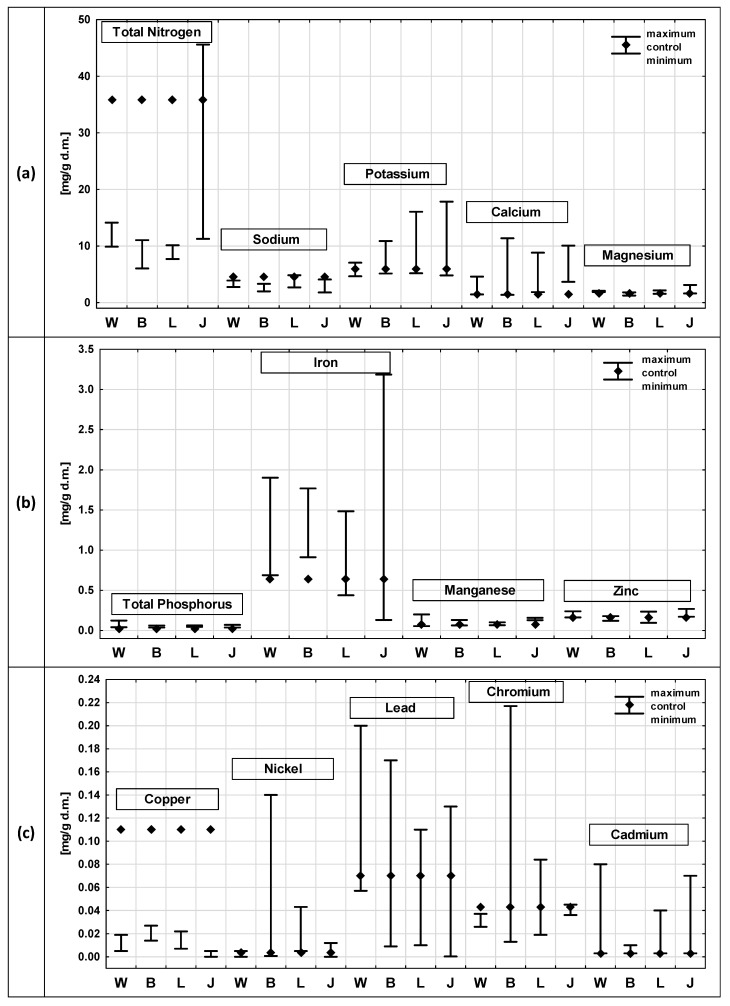
Comparison of chemical composition of plants after exposure to leachate from active (L—Legnica, J—Jawor) and closed (W—Wrocław, B—Bielawa) landfills with the composition of the control sample—results of analyses for *Phragmites australis* roots: (**a**) contents of TN, sodium, potassium, calcium and magnesium; (**b**) contents of TP, iron, manganese and zinc; (**c**) contents of copper, nickel, lead, chromium and cadmium.

**Figure 4:**
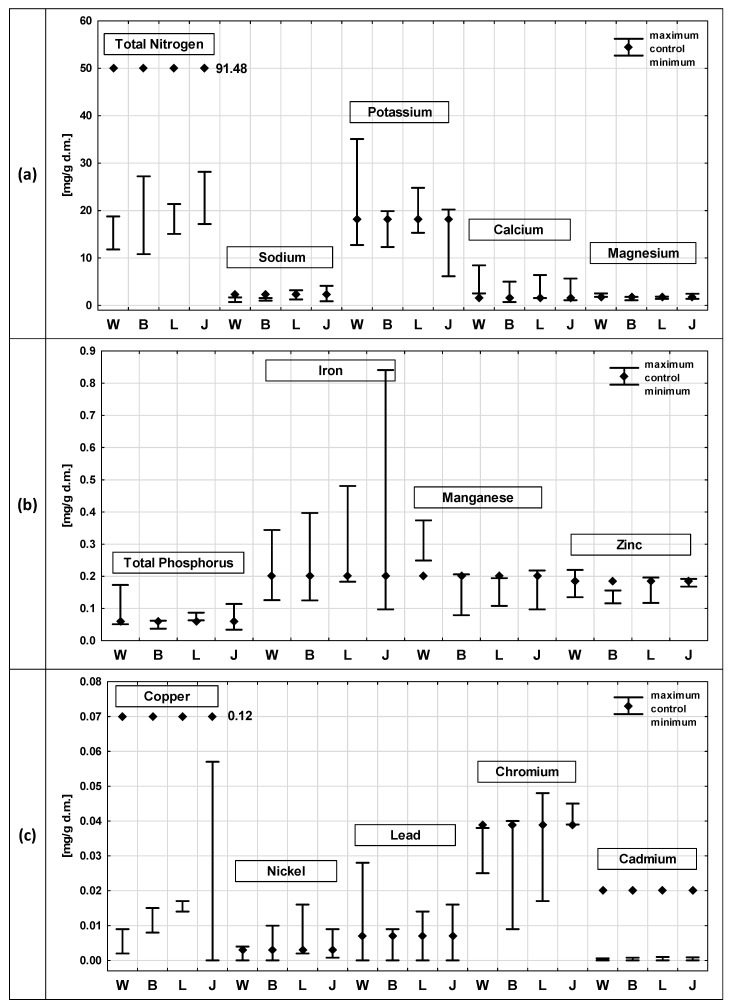
Comparison of chemical composition of plants after exposure to leachate from active (L—Legnica, J—Jawor) and closed (W—Wrocław, B—Bielawa) landfills with the composition of the control sample—results of analyses for *Phragmites australis* shoots: (**a**) contents of TN, sodium, potassium, calcium and magnesium; (**b**) contents of TP, iron, manganese and zinc; (**c**) contents of copper, nickel, lead, chromium and cadmium.

Figure 3 and Figure 4 show the results of the chemical composition analyses of *P. australis* after exposure to the leachate from the four municipal waste landfills and in the control samples, divided into roots (Figure 3) and shoots (Figure 4). The analysis of the chemical composition of the plants included content determinations of: TN, TP, sodium, potassium, calcium, magnesium, iron, manganese, zinc, lead and nickel, cadmium, copper and chromium.

The TN content was, in almost all cases, lower in the roots than in the shoots, except for the exposure to the Jawor leachate, where the maximum TN content in the root (45.6 mg/g) exceeded that observed in the shoots (28.2 mg/g). The highest TN content was recorded in the reed shoots in the control sample (91.5 mg/g) and it was more than 2.5 times higher than the value recorded in the roots.

In the case of Na, no such clear differentiation was found, and in all cases the contents did not exceed 4.13 mg/g. In the shoots of the control samples, three times higher Na contents were recorded than in the roots. Similarly, for the Ca content, the tendency was variable. The highest value was recorded in the roots after exposure to the leachate from the Bielawa landfill (11.4 mg/g) and from the Jawor landfill (10.1 mg/g). In the case of the shoots, the value was lower and amounted to 8.4 mg/g (in the leachate from the Wrocław landfill).

Higher K concentrations were observed in the *P. australis* shoots than in the roots. The highest K value was recorded in the *P. australis* shoots after exposure to the leachate from Wroclaw. The Mg content in the roots and shoots were similar, ranging from 1.08 mg/g (Bielawa, shoots) to 3.11 mg/g (roots, Jawor). A slightly higher value was recorded in the shoots in the control sample of *P. australis* than in the roots. No significant differences between the TP content in the roots and shoots were observed either; they ranged from 0.02 mg/g (roots, control sample) to 0.17 mg/g (shoots, Wrocław). In most cases, the TP content was higher in the shoots than in roots (except for the leachate from the Jawor landfill).

The Fe content in the *P. australis* roots were higher than in the shoots in each of the analysed cases. The values ranged from 0.1 mg/g (Bielawa, shoots) to 3.2 mg/g (Jawor, roots). According to the data presented in the literature, the roots and rhizomes of *P. australis* can accumulate higher amounts of heavy metals than the remaining organs, which is determined by the fact that they have large, airy intercellular spaces in the parenchyma [83].

The Mn contents were, in all cases, higher in the shoots than in roots, except for the Jawor landfill, where a slightly higher value was recorded in the roots. The Mn contents were low and did not pose any potential toxicity to the plants—they ranged from 0.063 mg/g to 0.37 mg/g. As reported in the literature, the toxic concentration of Mn for different plants ranges from 0.4 mg/g to 1 mg/g [84].

The Zn contents in the roots and shoots of *P. australis* were similar in all cases, ranging from 0.09 mg/g to 0.27 mg/g. Similar contents were reported by Vymazal et al. [85], where the Zn content in the *P. australis* roots ranged between 0.0135 mg/g and 0.202 mg/g dry mass.

Slightly higher values were observed in the roots than in the shoots, except for the control sample and the minimum value, recorded after exposure to the Legnica leachate. As reported in the literature, the toxic concentration of Zn for different plants ranges from 0.1 mg/g to 0.4 mg/g [84]. The Zn content of the leachate remained at levels that did not pose potential toxicity to *P. australis.*

The contents of the heavy metals (Cu, Ni, Pb, Cr and Cd) were low in all cases and did not exceed 0.06 mg/g. In most cases, higher metal contents were observed in the roots than in the shoots, but these differences were insignificant. In studies conducted on *P. australis*, it was proven that underground organs show a higher capacity to accumulate heavy metals compared to shoots [81]. The increased accumulation of heavy metals in underground organs may result, for example, from a defence mechanism that protects the species from the harmful effects of toxic concentrations on photosynthetic processes and prevents the translocation of toxic concentrations from roots to aboveground organs [86].

In the conducted studies, only in some cases a higher accumulation was observed in the roots, while in the remaining cases, similar metal contents were recorded in these parts of the plants.

As reported in the literature, the toxic concentration of Pb for different plants ranges from 0.01 mg/g to 0.1 mg/g [84]. *P. australis,* under natural conditions, grows on substrates where the heavy metal content does not exceed: Cd (<0.04 mg/g), Pb (<15.9 mg/g), Cu (<0.275 mg/g), Cr (<0.218 mg/g) and Ni (<0.082 mg/g) [65]. Common reed has the ability to take up large amounts of micronutrients due to its extensive tissue system and defence mechanisms [64]. The ability of plants to accumulate nutrients and toxic elements, including heavy metals, has been confirmed by numerous researchers [87,88]. It has been shown that higher aquatic plants are able to accumulate metal ions to levels well beyond their physiological needs, but excess can cause deleterious effects in plants [88].

The conducted analysis of the chemical composition of *P. australis* roots and shoots showed that the levels of Na, Mg, Zn and TP differed slightly in all the analysed variants. Most of the analysed parameters were higher in the external solution than in the *P.* *australis* roots. Higher concentrations in the external solution were observed in both *P. australis* and *C. demersum*, which can be justified by the fact that only a limited number of elements move easily, thus being absorbed by the plants [40,89]. However, it also happened that higher values were recorded in *P. australis* roots than in the external solution, e.g., for: Cr, Pb and Ni (after exposure to the leachate from Bielawa, Wrocław and Jawor) as well as Zn and Mn (after exposure to the leachate from Wrocław) and Fe (after exposure to the leachate from Bielawa). Additionally, in the case of the *P. australis* shoots, higher contents were recorded than in the external solution in several cases. Higher concentrations in shoots were recorded for the same parameters as in the roots, i.e., Fe, Mn, Zn, Cr, Pb and Ni. In addition, a several times higher concentration of TN was observed in the *P. australis* shoots after exposure to the leachate from the Wrocław landfill than in the external solution.

### 3.2. Analysis of Similarities between the Composition of C. demersum and P. australis

**Figure 5:**
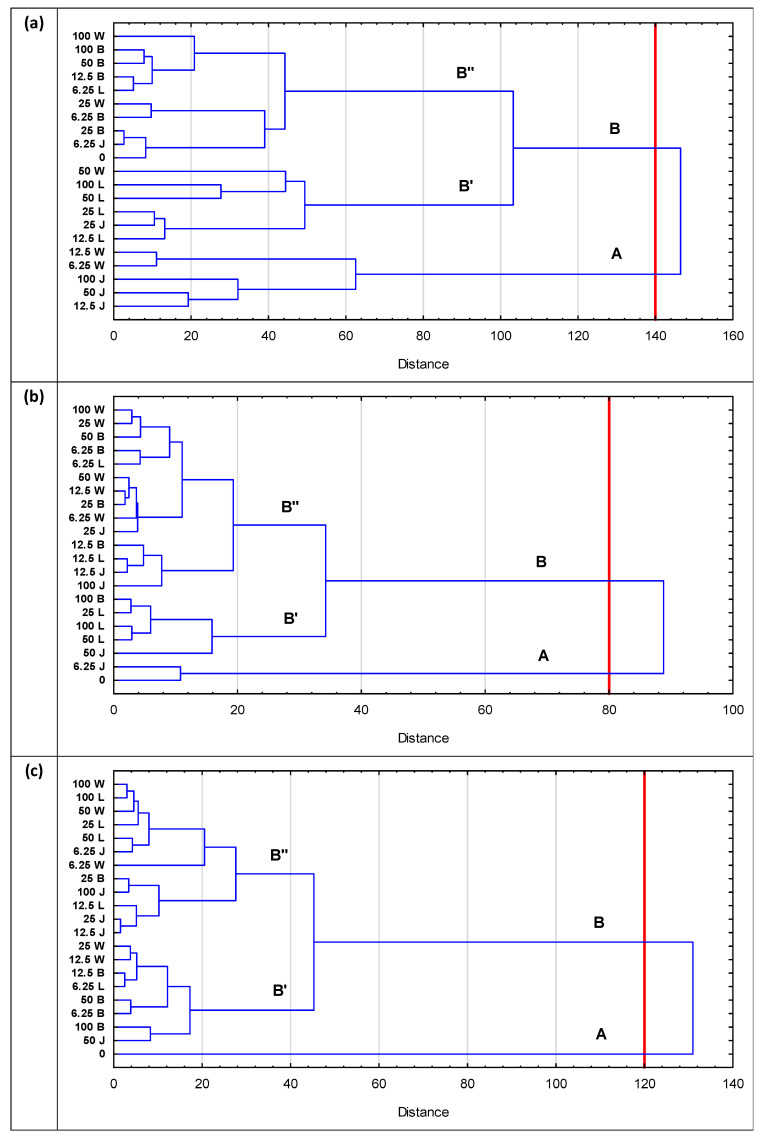
Dendrogram showing the similarities between the analysed samples: (**a**) *Ceratophyllum demersum*, (**b**) *Phragmites australis*—roots, and (**c**) *Phragmites australis*—shoots. Agglomeration was carried out using Ward’s method.

The assessment of the similarity between the chemical composition of the plant samples after the end of the exposure to different concentrations of leachate from the landfills included in the study was carried out using cluster analysis. The dendrogram diagram for the analysed samples of rigid hornwort (Figure 5a) shows two main clusters (A and B). Cluster A includes only samples of plants treated with the leachate from the Wrocław and Jawor landfills. The greatest similarity is between the samples 6.25 and 12.5 W and 12.5, 50 and 100 J, which form the smallest clusters. Cluster B is divided into B’ and B”. Cluster B’ is dominated by the samples of the plants treated with the leachate from the Legnica landfill. The smallest clusters are formed by the samples 25 L, 25 J and 12.5 L and 50, 100 L and 50 W. Cluster B” includes all the samples of plants treated with the leachate from the landfill in Bielawa and also the control sample (0). The highest similarity is found between the samples 25 B, 6.25 J and 0, 6.25 B and 25 W and between the samples 12.5B, 6.25 L, 50 and 100 B and 100 W. The analysis of the clusters forming the dendrogram for the examined rigid hornwort samples shows a fairly clear influence of the source of origin of the treated leachate (landfill) on the formation of individual clusters. The similarity between the composition of the plants treated with the most diluted leachate from the oldest, non-operational landfill in Wrocław and the plants subjected to the highest concentrations of the leachate from the Jawor landfill, active during the study, is also evident. The low degree of the contamination of the leachate from the Jawor landfill is confirmed by the similarity between the plants treated with the sample with the lowest concentration and the control sample, shown in the figure. The samples of the plants treated with the leachate from the other landfills (in Bielawa and Legnica) were found in separate clusters and the distances between those clusters prove high differences in the composition of those samples.

Figure 5b shows a dendrogram illustrating the results of the cluster analysis for the reed root samples used to treat the different concentrations of leachate from the four landfills included in the study. On the basis of similarities, the samples were divided into two main clusters (A and B). Cluster A includes the control sample (0) and the roots of the reed used to treat the leachate from the Jawor landfill at the lowest concentration (6.25 J). All the other samples differed from the two mentioned above significantly enough to be included in the second cluster (B). Cluster B is divided into B’ and B”. Cluster B’ mainly includes samples of the reed roots used for the treatment of the leachate from the Legnica landfill (25, 50 and 100 L) as well as the samples of 100 B and 50 J. The biggest part of cluster B’ are the samples of the reed roots used for the treatment of the leachate from the Wrocław landfill (all) and the samples for the Bielawa landfill (except for 100 B). The presented dendrogram illustrates clear differences between the control sample and the reed root samples used for landfill leachate treatment. A difference is also evident between the majority of the samples for the Legnica landfill (25, 50 and 100 L, cluster B’) and the remaining samples, mainly corresponding to the Wrocław and Bielawa landfills (cluster B”).

Figure 5 c shows the results of the cluster analysis for the reed shoot samples used to treat the different concentrations of leachate from the four landfills included in the study. The largest differences were between the control sample (0, cluster A) and the other samples (cluster B). In cluster B, there are also two smaller differing samples (B’ and B”). Cluster B’ mainly includes samples of the reed shoots used for treating the leachate from the Bielawa landfill (except for 25 B) and corresponds to the lower concentrations of the leachate from the landfills in Wrocław (12.5 and 25 W), Legnica (6.25 L), and also sample 50 J. Cluster B” consists mainly of the samples of the reed shoots used for treating the leachate from the landfills in Legnica (100, 50, 25 and 12.5 L), Jawor (100, 25, 12.5 J) and Wrocław (6.25, 50 and 100 W). The presented dendrogram illustrates in particular the clear difference between the samples of the reed shoots used for the landfill leachate treatment and the control sample (0). This difference is even greater than in the analysis performed for the reed roots, which is related to the placement of the cut-off level (defining the number of significant clusters) at a greater distance. The remaining clusters show a greater mixing of samples, which indicates the occurrence of similarities in chemical composition.

### 3.3. Bioconcentration Factor (BCF)

The bioconcentration factor (BCF) determines the ability of a plant to accumulate elements from the substrate [90]. Table 2 shows the calculated bioconcentration factor (BCF) values for two plants, i.e., *Phragmites australis* and *Ceratophyllum demersum*, after exposure to the leachate from the four municipal waste landfills and in the control samples.

The (BCF) values in the presented studies ranged from 0 to 10^1^. A BCF above one is a key feature of hyperaccumulators [91,92]. The BCF can reach different orders of magnitude. Maderra-Parra et al. [31], in studies on the phytoremediation of landfill leachate, obtained BCFs ranging from 10^0^ to 10^2^. Pandey et al. [82] obtained similar BCF values. It is reported in the literature that a good bioaccumulator should exhibit a BCF above 1000 [93,94].

The BCF for *P. australis* and *C. demersum*, after exposure to the leachate from the landfills in Legnica and Jawor, was <1 in each variant. However, in the case of the other two landfills, i.e., Bielawa and Wrocław, the BCF values above one were obtained only for Cr for *P. australis* and TN for *C. demersum*.

In the conducted studies, for *C. demersum*, the BCF for Cr was <1 in all cases. However, Abdallah [46], in a study on the phytoremediation of metals from aqueous solutions by *C. demersum,* proved that it is a good accumulator of Cr, obtaining BCF values > 1000 in all cases. The BCF for Cu in all cases for *P.australis* was below one. However, after the exposure of *C. demersum*, the BCF for Cu only in the control sample was above one (i.e., BCF—4.59). The highest BCF value for *Ceratophyllum demersum* was recorded for Mn in the control sample and it amounted to 32.25. A higher BCF value was also obtained for Fe after exposure to the leachate from the Wrocław landfill (BCF—23.91).

In the case of the *Phragmites australis* exposure, the highest BCF value was obtained for TN in the control sample (BCF—12.74). Similar values to those obtained in this study were obtained by Sochacki et al. during a study conducted on *P. australis*, where the BCF ranged up to 10^1^ and they concluded that this species does not play an important role as a metal accumulator [95]. On the other hand, Daud et al. [32], investigating the potential of *Lemna minor* in the phytoremediation of landfill leachate, obtained much lower BCF values; in each case, these were less than one. The discrepancies in the BCF values obtained indicate that different plant species develop different mechanisms for tolerating or accumulating heavy metals, depending on specific environmental conditions [96]. In turn, the ability to accumulate heavy metals in plants depends on their ability to uptake and transport intracellularly, which includes, inter alia, mobilization in the rhizosphere and the transport of metals across the plasma membrane of root cells [97].

It is reported in the literature that submergent plant species are able to accumulate a higher amount of heavy metals compared to emergent species due to the fact that they can remove heavy metals from the water with their entire surface [21,98]. However, no significant differences were observed between *C. demersum* and *P. australis* in the study conducted. Additionally, Vymazal et al. [85] did not observe any significant differences between the plants, and the element concentrations found in both plants (*Phragmites and Phalaris*) were at very similar levels.

### 3.4. Translocation Factor (TF)

**Figure 6:**
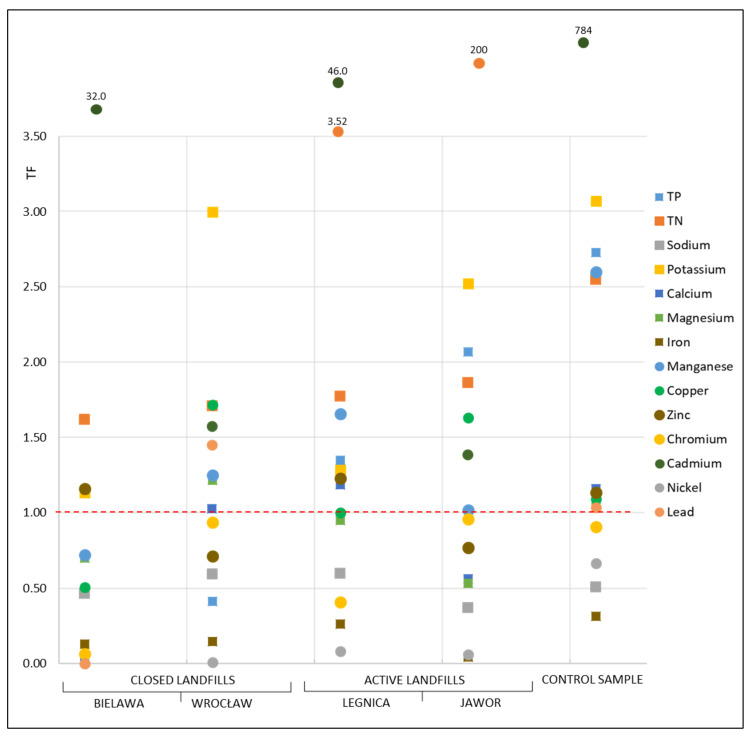
Translocation factor (TF) of pollutants of *Phragmites australis* in leachate from four municipal waste landfills.

The ability to translocate pollutants in different parts of the plant can be assessed using the translocation factor (TF). Figure 6 shows the calculated translocation factor (TF) values for *P. australis* after exposure to the leachate from the four municipal waste landfills and in the control sample.

The uptake and translocation of individual components by plants from water or soil, in addition to their availability, are also influenced by ongoing reactions (antagonistic and/or synergistic) between individual elements [81]. A shoot:root translocation ratio (S/R) greater than one indicates the efficient transport of components from roots to shoots and is a key feature of all hyperaccumulators [99]. In contrast, a TF below one indicates that pollutants are accumulated in the lower part of the plant [100].

The translocation factor varies depending on the type of pollutant [101], as confirmed by the analyses performed. Its magnitude is also influenced by the plant species. It has been observed that some plant species store components in roots (i.e., root accumulators) and some in shoots (i.e., shoot accumulators). The translocation factor may also be influenced by changes in the epidermis and mesoderm of plants, e.g., caused by an increased content of metals, which hinders the migration of nutrients from the roots to the shoots [97].

In the case of Na, Fe, Cr and Ni, no transposition into the aboveground parts of *P. australis* was observed, either after exposure to the leachate from both active and closed facilities or in the control sample. The low TF for Na, Fe, Cr and Ni in the aboveground parts of *P. australis* can be explained by the absorption and/or adsorption of ions from the aqueous solution through the roots, as the shoots under normal conditions do not play such a role [92]. The low TF values for nickel can be further justified by the fact that nickel is preferentially adsorbed by mineral components [102]. Additionally, Bonanno et al. [81] observed that Cr shows low mobility from roots to shoots in *P. australis* tissues. It is also reported in the literature that Fe shows high accumulation in roots but low translocation from roots to the remaining organs [40], which is consistent with the results obtained in the present study.

Other authors, during studies conducted on hydrophytic species, also found no or a minimal transposition of components to the aboveground parts of plants, observing the highest accumulation of metals in the roots [24,25]. Stoltz and Greger [103], in studies conducted, among others, on *P. australis,* observed that most of the micronutrients they studied (i.e., Cd, Cu, Zn and As) accumulated only in the plant roots and did not move to the shoots, with the exception of Pb, which accumulated both in the roots and shoots. Additionally, Bonanno and Giudice [81], during their study on the bioaccumulation of metals (i.e., Cd, Cr, Cu, Hg, Mn, Ni, Pb and Zn) by *P. australis,* showed low mobility from the roots to the aboveground organs, proving that underground organs are the main zones of metal accumulation. It has been observed in many studies that the concentration of elements in the roots is much higher than in the leaves and shoots, which confirms that the roots are the main site for metal uptake [104].

In the presented study, only in the case of cadmium transposition to the aboveground parts of *P. australis* was recorded in each of the analysed variants. The highest TF for cadmium was recorded in the control sample, where it was 784.

In the case of Mn, a low TF was recorded only after exposure to the leachate from the closed landfill in Bielawa, which means accumulation in the roots. In all other cases, the movement of Mn from the roots to the shoots was observed.

The Zn accumulated both in the roots (in the case of exposure to the leachate from the landfills in Wrocław and Jawor) and moved to the aboveground parts (in the case of exposure to the leachate from the landfill in Bielawa, Legnica and the control sample).

## 4. Conclusions

The analysis of micro- and macroelements in two selected plants, i.e., emerging macrophytes (*P. australis*) and submerged macrophytes (*C. demersum*), treated with leachate from four municipal landfills, showed that:

1. The concentrations of the heavy metals (Cu, Ni, Pb, Cr and Cd) in the studied plants were low in all the analysed cases. Higher metal contents could often be observed in the roots rather than in the shoots, but these differences were insignificant.
2. The chemical composition of the studied plant samples was primarily related to the source of origin of the treated leachate (landfill), as clearly demonstrated by cluster analysis for the *C. demersum* samples. However, in the case of the roots and shoots of *P. australis*, significant differences were observed between the control and leachate-treated samples. In many cases, similarities were found between plant samples after exposure to different leachate concentrations, which was due to minor differences in their composition.
3. The conducted studies showed that *P. australis* was not effective in translocating certain elements (i.e., Na, Fe, Cr and Ni). The TF in all cases was below 1.0, indicating that translocation with *P. australis* may not be an effective option for removing high concentrations of pollutants contained in leachate. Cadmium showed the highest translocation to the aboveground parts of *P. australis* (in each of the analysed variants).
4. The bioconcentration factor BCF, for the majority of the analysed parameters in *P. australis* and *C. demersum*, remained at a low level. It reached values >1 only in a few cases, which demonstrates that both plants do not exhibit the features of a good hyperaccumulator of the pollutants contained in leachate.
5. In the conducted studies, no significant differences were observed in the accumulation of the studied components between submergent plants (*C. demersum*) and emergent macrophytes (*P. australis*).

## Figures and Tables

**Table 1:** Physicochemical characteristics of raw landfill leachate.

Pollution Indicators	Unit	Non-Operational Landfills		Active Landfills	
		Bielawa	Wrocław	Legnica	Jawor
pH	-	8.42	8.16	8.84	8.11
EC	μS/cm	2318	1800	7791	3919
TKN	mg N/dm^3^	51.12	1.09	269.85	310.16
TP	mg P/dm^3^	3.11	0.22	3.936	12.04
Sodium	mg Na/dm^3^	151.8	91.2	177.8	162.9
Potassium	mg K/dm^3^	256.2	61.2	507.6	410
Calcium	mg Ca/dm^3^	150.3	309.4	68.1	171.5
Magnesium	mg Mg/dm^3^	79.3	52.5	87.9	91.2
Iron	mg Fe/dm^3^	0.5	0.15	2.16	3.81
Manganese	mg Mn/dm^3^	0.35	0.02	0.47	1.61
Copper	mg Cu/dm^3^	0.0457	0.0486	0.1448	0.059
Zinc	mg Zn/dm^3^	0.1977	0.2241	0.4987	0.3049
Chromium	mg Cr/dm^3^	0.00025	0.00025	0.1927	0.00025
Lead	mg Pb/dm^3^	0.0163	0.0054	0.0866	0.004
Nickel	mg Ni/dm^3^	0.00025	0.00025	0.1716	0.0209
Cadmium	mg Cd/dm^3^	0.0033	0.0047	0.0033	0.0039

**Table 2:** Bioconcentration factor (BCF) for *Phragmites australis* and *Ceratophyllum demersum* (for *P. australis*, the average for roots and stems was taken).

Pollution Indicators	*Phragmites australis*					*Ceratophyllum demersum*				
	Active Landfills		Non-Operational Landfills		Control Sample	Active Landfills		Non-Operational Landfills		Control Sample
	Legnica	Jawor	Bielawa	Wrocław		Legnica	Jawor	Bielawa	Wrocław	
TP	0.00	0.00	0.00	0.00	0.04	0.00	0.00	0.00	0.00	0.09
TKN	0.00	0.00	0.00	0.01	**12.74**	0.00	0.00	0.00	0.01	**8.13**
Sodium	0.00	0.00	0.00	0.00	0.00	0.00	0.00	0.00	0.00	0.00
Potassium	0.00	0.00	0.00	0.00	0.01	0.00	0.00	0.00	0.00	0.03
Calcium	0.00	0.00	0.00	0.00	0.00	0.00	0.00	0.00	0.00	0.00
Magnesium	0.00	0.00	0.00	0.00	0.00	0.00	0.00	0.00	0.00	0.00
Iron	0.00	0.00	0.01	0.01	0.21	0.00	0.00	0.01	**23.91**	0.40
Manganese	0.00	0.00	0.00	0.00	**5.59**	0.00	0.00	0.01	0.01	**32.25**
Copper	0.00	0.09	0.60	0.30	**4.59**	0.00	0.01	0.00	0.02	0.80
Zinc	0.00	0.00	0.00	0.00	0.00	0.00	0.00	0.00	0.00	0.00
Chromium	0.00	0.00	**4.62**	**1.02**	**1.65**	0.00	0.00	0.45	0.41	0.64
Cadmium	0.00	0.00	0.00	0.00	0.39	0.00	0.00	0.00	0.00	0.00
Nickel	0.00	0.00	0.00	0.00	0.00	0.00	0.00	0.00	0.00	0.00
Lead	0.00	0.00	0.00	0.00	0.00	0.00	0.00	0.00	0.00	0.00

BCF values above 1 are shown in bold in the table.

## Data Availability

Not applicable.
